# Standardized Ileal Digestibility of Calcium and Phosphorus in Feed Ingredients for 21-Day-Old Broilers

**DOI:** 10.3390/ani14172603

**Published:** 2024-09-07

**Authors:** Chae-Won Lee, Changsu Kong

**Affiliations:** 1 Department of Animal Science and Biotechnology, Kyungpook National University, Sangju 37224, Republic of Korea; chaewon2991@gmail.com; 2 Department of Animal Science, Kyungpook National University, Sangju 37224, Republic of Korea; 3 Research Institute for Innovative Animal Science, Kyungpook National University, Sangju 37224, Republic of Korea

**Keywords:** broiler, calcium, feed ingredient, ileal digestibility, phosphorus

## Abstract

**Simple Summary:**

Plant-based ingredients are the major constituents of poultry diets and supply a significant portion of dietary phosphorus (P). However, approximately two-thirds of the P of plant origin is bound to phytic acid, which is poorly available to monogastric animals. Broiler diets have been formulated based on non-phytate P (NPP), which might lead to the excessive use of inorganic P sources and reduced P availability for birds. Recent research has suggested using digestible P to assess availability, and due to nutritional interactions, there is also increasing interest in determining calcium (Ca) digestibility. The objectives of this study were to determine the standardized ileal digestibility (SID) of Ca and P in the feed ingredients for broilers. On day 18 post-hatch, 512 male and female broilers were individually weighed and randomly allocated to eight treatments with four replicates for each sex (eight birds/cage) in a randomized complete block design based on body weight and sex. No significant interaction between experimental diets and sex regarding the SID of Ca or P was found, and no effect of sex on the SID was observed. There were significant differences in the SID of the feed ingredients.

**Abstract:**

This study aimed to determine the standardized ileal digestibility (SID) of calcium (Ca) and phosphorus (P) in various feed ingredients using the direct method. This study comprised eight experimental diets: a Ca–P-free diet and seven experimental diets, each containing monocalcium phosphate (MCP), dicalcium phosphate (DCP), monosodium phosphate (MSP) + limestone, corn, and soybean meal (SBM) as the sole sources of Ca and/or P. These diets provided 4.21 g/kg of non-phytate P from MCP, DCP, or MSP, and the MSP + limestone diet included 7.50 g/kg of Ca. The corn and SBM diets formulated to determine P digestibility maintained a dietary Ca/total P ratio of 1.4 through the addition of limestone. Chromic oxide was added to the diets as an indigestible index. On day 18, 256 male and 256 female broilers were individually weighed and randomly assigned to eight treatments, each with four replicates for each sex (eight birds per cage). This allocation followed a randomized complete block design based on body weight. On day 21, the birds were euthanized using carbon dioxide, and ileal digesta samples were collected from the distal two-thirds section of the ileum. No significant interactions between the experimental diets and sex regarding the SID of Ca or P were detected, and no effect of sex on the SID was observed. The standardized ileal Ca digestibility of MCP, DCP, limestone, corn, and SBM was found to be 84.7%, 70.1%, 52.6%, 88.6%, and 81.6%, respectively. The standardized ileal P digestibility of MCP, DCP, MSP, corn, and SBM was determined to be 91.8%, 76.8%, 94.4%, 73.1, and 88.4%, respectively. Given the variable digestibility of Ca and P across different feed ingredients, the consideration of the specific type of ingredients used in diet formulation is crucial.

## 1. Introduction

Calcium (Ca) and phosphorus (P) are the most abundant minerals in the body and are essential for the formation and maintenance of the skeleton [1]. In plant seed-based poultry diets, the dietary Ca and P contents are commonly supplemented with inorganic sources or animal-derived ingredients to meet the poultry requirements. Dietary phosphates are typically acquired from rock phosphate [2]. Monocalcium phosphate (MCP), dicalcium phosphate (DCP), and monosodium phosphate (MSP) are often used to supply dietary P and a portion of dietary Ca, while limestone is the major Ca source in poultry diets [3,4,5]. Corn and soybean meal (SBM) contain relatively lower concentrations of Ca and P compared to inorganic sources; however, they are the major feed ingredients for energy and protein sources in the diet.

Poultry diets are commonly formulated based on the total Ca and available P ([avP], [6]). The use of dietary Ca has not transitioned toward a digestible Ca system [7,8], and this may be attributed to the relatively low cost of limestone compared with that of other mineral sources in poultry diets [3,4,9]. However, dietary P is often supplied based on avP, which is defined as the portion of dietary total P that can be utilized to meet P requirements [10]. Non-phytate P (NPP) is commonly used interchangeably with avP [1,7,11], although avP encompasses both digestible inorganic and organic P [12,13]. Mutucumarana et al. [14] reported that the digestible P content in corn and canola meal exceeded that of NPP, indicating that broiler chickens utilized a portion of the phytate-bound P. Hence, avoiding the interchangeable use of these terms is essential, as it potentially leads to inaccuracies. Li et al. [13] suggested the use of quantitative values, such as retainable P and pre-cecal digestible P, to measure avP. However, once a physiological threshold is reached, P excretion via urine increases [14]. Therefore, to exclude the contribution of urinary P excretion, ileal digestibility is preferred when determining the availability of feed ingredients [15,16].

The determination of the ileal digestibility of Ca and P has yielded varying results across various studies using different methodologies. The direct method requires only one diet using a test ingredient as the sole source of the interest component and renders it possible to obtain basal endogenous loss intuitively. Due to the influence of basal endogenous losses (BELs) on apparent ileal digestibility (AID), using standardized ileal digestibility (SID), which can be calculated using corrected AID values for endogenous loss, is recommended to ensure the validity of the additive assumption [17]. The concentrations of Ca and P in diets influence each other’s digestibility; however, most studies have measured the digestibility of only one of these minerals. Therefore, this study aimed to simultaneously determine the SID of Ca and P in various feed ingredients using the direct method.

## 2. Materials and Methods

Experimental procedures were reviewed and approved by the Institutional Animal Care and Use Committee of Kyungpook National University, Republic of Korea (approval number: KNU 2023-0183).

### 2.1. Animals and Experimental Design

Day-old Arbor Acres male and female broilers were initially housed on the floor in an environmentally controlled room from day 0 to 10 and subsequently transferred to a battery cage on day 10. On day 18, a total of 256 males and 256 females were individually weighed and assigned to eight treatments, each with four replicates for each sex in a randomized complete block design (eight birds per cage). During the experimental period, the room temperature was maintained at 27 °C, and 24 h lighting was provided. The birds had ad libitum access to diets and water from nipple drinkers.

### 2.2. Dietary Treatments

The experimental treatments comprised a Ca–P-free diet and seven semi-purified diets. These diets included MCP, DCP, MSP + limestone, corn, and SBM as the sole sources of Ca and/or P. The diets containing MCP, DCP, and MSP + limestone provided 4.21 g/kg of NPP. The MSP + limestone diet included 7.50 g/kg of Ca. To assess ileal Ca digestibility, corn and SBM diets were formulated with P concentrations adjusted using MSP to attain a P concentration of 4.21 g/kg. The Ca/total P ratio was maintained at 1.4 by adding limestone to evaluate P digestibility in corn and SBM. All experimental diets were provided in mashed form for 3 days, and essential amino acids of these diets met or exceeded the requirement recommended by the Arbor Acres broiler nutrition specifications [18]. Chromic oxide (5 g/kg of diet) was incorporated into all diets as an indigestible index.

### 2.3. Sample Collection

On day 21, all the birds were euthanized using CO_2_ asphyxiation to enable the collection of ileal digesta. Ileal digesta were collected from the distal two-thirds section of the ileum, between Meckel’s diverticulum and the ileocecal junction, by flushing with distilled water. The collected samples were then immediately stored at −20 °C for preservation. Subsequently, the digesta samples were dried in a forced-air oven (JSOF-150; JS Research, Gongju, Korea) at 55 °C, and all experimental diets and digesta samples were ground using a mill grinder (CT 293 Cyclotec; Foss, Eden Prairie, MN, USA) for further analysis.

### 2.4. Chemical Analyses and Calculations

The experimental diets were analyzed in duplicate for dry matter using a forced air-drying oven (method 930.15) [19] to calculate the BEL. The chromium concentrations in the experimental diets and ileal digesta samples were determined using the method described by Fenton and Fenton [20]. Ca and P concentrations were analyzed using procedures prescribed by the Association of Official Analytical Chemists (method 968.08) [19]. The AID, BEL, and SID of Ca were calculated using the following equations [21]:

AID (%) = 100 − [(Cr_diet_/Cr_digesta_) × (Ca_digesta_/Ca_diet_)] × 100 (1)

BEL (mg/kg of DM intake) = (Cr_diet_/Cr_digesta_) × Ca_digesta_, (2)

SID (%) = AID + (BEL/Ca_diet_) × 100, (3)

where Cr_diet_ and Cr_digesta_ represent the chromium concentrations (mg/kg) in the experimental diet and ileal digesta samples, respectively, while Ca_diet_ and Ca_digesta_ represent the Ca concentrations (mg/kg) in the experimental diet and ileal digesta samples, respectively. The AID, BEL, and SID of P were calculated using the same equations by substituting Ca with P.

### 2.5. Statistical Analyses

Data were analyzed using the MIXED procedure of SAS software 9.4 (SAS Inst. Inc., Cary, NC, USA). The dietary treatments were evaluated for the main effects, whereas the block and sex were considered random variables. The cage served as the experimental unit. Least squares means were calculated, and statistical differences were considered at *p* < 0.05. If dietary effects were significant, Tukey’s adjustment was used to compare mean values between the treatments.

## 3. Results

The calculated and analyzed composition of the experimental diets are presented in Table 1. The analyzed concentrations of Ca and total P were all close to calculated values, except for the diets containing SBM. Total P concentrations in the diets were higher than the calculated values by 1.54–1.97 g/kg. The analyzed composition of the feed ingredients is shown in Table 2. Broilers remained healthy during the experimental period, and no mortality or leg problems were recorded. Regarding the SID of Ca and P, no significant effect of sex on the SID was observed (*p* > 0.05). There were significant differences in the digestibility of the experimental diets (*p* < 0.05). The SID of Ca and P for MCP, DCP, the mixture of MSP and limestone, corn, and SBM are shown in Table 3. The ileal endogenous losses of Ca and P were determined to be 58.98 and 66.37 mg/kg of dry matter intake (DMI), respectively, and were used to calculate the SID. The results revealed that the SID of Ca ranged from 52.6% to 88.6 with the highest values observed in corn, followed by MCP, SBM, DCP, and limestone. The SID of P with the highest values were observed in MSP, followed by MCP, SBM, DCP, and corn.

## 4. Discussion

Inorganic P is a non-renewable resource, and excessive P excretion can cause an unnecessary waste of resources and environmental pollution. The precise determination of dietary P digestibility in poultry diets is required to optimize dietary Ca and P concentrations to meet the requirements of birds. This optimization aims to enhance the efficiency of Ca and P utilization in diets. When assessing Ca and P digestibility, both minerals are commonly considered together, because Ca and P interact in the gastrointestinal tract [22]. For instance, P absorption is known to increase as dietary Ca concentration decreases [23]. Furthermore, an imbalanced Ca:P ratio may aggravate growth performance and bone mineralization [24]. It can also contribute to the formation of an insoluble phytate–Ca complex, potentially reducing the availability of phytate P [11,25]. However, a standardized quantity of Ca or P to be incorporated in digestibility assessments is yet to be established. Based on the observations from previous studies, broilers can adapt to wide dietary Ca concentrations [13,26]. Therefore, in this study, the dietary NPP concentration in the experimental diets was formulated to meet the requirements recommended by the breeder company [18], and the SID of Ca or P was concurrently measured within the experimental diet.

The analyzed total Ca and P concentrations in the experimental diets were close to the calculated values, except for the total P concentration in the experimental SBM-containing diets. Nevertheless, the P content is deemed to have originated from SBM and is unlikely to have influenced the digestibility measurements. Therefore, the analyzed values were utilized in digestibility calculations. The birds were healthy during the experimental period, and no mortality or leg problems were recorded.

Sex was initially set as a fixed variable, and the digestibility for each sex was calculated separately. Sex did not affect the SID of Ca and P, and this is consistent with the findings of Mirabile et al. [27], wherein no difference in the SID of P was observed between male and female broilers. Conclusively, sex was considered as a random effect, hence the replication of digestibility was set to eight.

The BEL of Ca and P was measured in the Ca–P-free diet and was found to be 58.98 and 66.37 mg/kg of dry matter intake (DMI), respectively, and the values were within the range (29 to 236 and 25 to 438 mg/kg of DMI, respectively) of previously reported values [3,28,29,30,31].

The SID of Ca in feed ingredients varies widely depending on the experimental method. David et al. [32] determined a true ileal Ca digestibility of 43% and 32% for MCP and DCP, respectively. This indicates that the SID of Ca in MCP was higher than that in DCP and is consistent with the current study. However, the Ca digestibility was lower than current observations, and the different composition of the experimental diet could influence the Ca digestibility. In the research above, dietary Ca content was maintained above the recommended value specified by the Ross 308 recommendation [33], which also exceeded that in the present study. Digestible Ca or P is assumed to be altered under deficient conditions, by increasing absorption rates and utilization efficiency to satisfy the requirement [7,34,35]. However, Zhang and Adeola [36] reported that the true ileal Ca digestibility values of DCP and limestone determined via the regression method were 67.1% and 63.7%, respectively. The authors indicated that no differences in the AID of Ca were observed in response to an increased Ca level when experimental diets were formulated to contain graded Ca levels (3.3, 4.3, and 5.3 g/kg) at a constant Ca:NPP ratio of 1.1:1. To minimize the impact of Ca and/or P deficiencies or excess on Ca digestibility, the determination of the Ca and P concentration should be prioritized. The determination of digestibility at different levels of Ca and P can contribute to establishing appropriate ranges of Ca and P concentrations for digestibility assessments. Other reasons for discrepancies among the studies could be due to differences in methodology. Anwar et al. [3] compared different methodologies and found that the direct method yielded higher Ca digestibility in DCP (0.34) than in the difference (0.21) and regression (0.13) methods. Lamp et al. [6] measured the AID of Ca for MCP (62.5%) and DCP (44.8%) in broilers aged 21 days, which is the same age as that of the broilers used in the current study. However, the adaptation period was longer (day 1 to 21) than that in the present study, and approximately 1.4 g/kg of limestone was supplemented in the experimental diets. Due to the differences in experimental methods, it becomes difficult to directly compare the results across the studies.

The Ca:NPP ratio of the limestone diet in the current study was 1.78, and Anwar et al. [3] found that the true ileal Ca digestibility coefficients of limestone were 0.63 and 0.56 at a Ca:NPP ratio of 1.5 and 2.0, respectively. In the study by Davie et al. [23], the apparent ileal Ca digestibility coefficients of limestone of two different origins were 0.50 and 0.43. The findings of previous studies aforementioned are similar to the present study. However, ileal Ca digestibility in limestone can vary depending on the dietary P content, and as the gastrointestinal tract develops, bird age can also impact the utilization of nutrients [37,38].

The ileal Ca digestibility of corn and SBM in broiler diets, as determined using the direct method, has been insufficiently investigated to enable comparison with the digestibility observed in the current results. The regression method has been employed as an alternative to estimate the ileal digestibility if applying the direct method is impractical [39]. In the present study, the regression method was presumed not to yield meaningful digestibility results as the differences in dietary Ca concentration among the assay diets, particularly the corn diets, were considered too narrow. Since the regression method is based on a linear relationship, the large differences in the digestible Ca between the assay diets are necessary [40]. In the present experiment, the analyzed Ca concentration in the corn was 0.3 g/kg. Corn is typically considered to contain a negligible amount of Ca, rendering it a suitable base for Ca-free diets [32]. Corn and SBM are the major ingredients of poultry diets with low dietary Ca concentrations. The present study revealed that the SID of Ca was 88.6% for corn and 81.6% for SBM, and these values are higher than that in DCP and limestone. Generally, corn and SBM constitute a significant proportion in poultry diets. Therefore, when considering the values based on standardized ileal digestible Ca values, the contribution of corn and SBM to the SID of Ca in the diet is likely to increase, although this may vary depending on the addition of mineral supplement sources. Therefore, further research on the Ca digestibility of plant-based ingredients is required to accurately calculate the standardized Ca concentration in feed ingredients. The low Ca concentration in test ingredients potentially increases the contribution of the BEL of Ca to the digesta. Therefore, the high SID of Ca in corn observed in the present study is speculated to be attributed to the BEL. For the SBM, a lower true ileal Ca digestibility coefficient of 0.51 was reported by David et al. [23] using 21-day-old birds. The total P concentration was similar to that in the current study; however, their dietary NPP concentration was lower than that in the present study, which included MSP as a source of dietary P. The increased Ca:NPP ratio due to the addition of MSP in the current study may contribute to the disparity in the results between the previous study and the present study.

A previous study conducted in our lab in a similar experimental environment exhibited comparable values, yielding a SID of P of 89.8% and 79.5% for MCP and DCP, respectively [41]. The variation in P digestibility assessed through a ring test described by Rodehutscord et al. [42] suggested the importance of a detailed standard methodology. Several studies have evaluated the SID of P in inorganic ingredients. Using the regression method, Shastak et al. [15] yielded a P digestibility of 30% and 67% for DCP and MSP in 21-day-old birds, respectively. Bikker et al. [43] reported a P digestibility of 78.3% and 59.0% for MCP and DCP, respectively. Trairatapiwan et al. [11] estimated P digestibility values of 69.3% and 64.6% for MCP and DCP, respectively, using the regression method. However, in a study by Cambra-López et al. [44], the P digestibility values of MCP and DCP were determined to be 87.4% and 86.6%, respectively, using the regression method. The variation in P digestibility across various studies and different laboratories highlights the need to standardize methods used for measuring P digestibility.

In the current study, each feed ingredient served as the sole source of P to determine the SID of P using the direct method. An additional P supplement is normally considered to meet the recommended requirement; however, to measure the SID of P in corn and SBM, it was impossible. Therefore, a dietary Ca/total P ratio of 1.4 was maintained by adding limestone. Using the regression method, Trairatapiwan et al. [45] generated P digestibility values of 33.8% and 42.3% for corn and SBM, respectively, and these values were lower than those found in the current study. In another study, Mutucumarana et al. [14,46] yielded a P digestibility of 67.6% and 79.8% for corn and SBM, respectively. Additionally, Mutucumarana et al. [47] reported different P digestibility coefficients of corn (0.73 and 0.43) and SBM (0.74 and 0.52) depending on the assay methodology used on 28-day-old broilers. In Method 1, the dietary Ca:NPP ratio was adjusted to 2:1 by adding limestone. In Method 2, the dietary Ca/total P ratio of 1.3:1 was achieved via limestone supplementation. The authors speculated that one of the reasons for the difference in digestible P contents between the methods could be the lower dietary Ca concentration in Method 1 than that in Method 2. The current study maintained a Ca/total P ratio of 1.4 in the corn and SBM diets, and the low Ca concentrations in current experimental diets might increase the SID of P. The study by Perryman et al. [26], reporting that the true ileal P digestibility is higher at a low Ca concentration (0.13%) compared to a high Ca concentration (0.95%), could support this observation.

## 5. Conclusions

In conclusion, the SID of Ca in MCP, DCP, limestone, corn, and SBM were determined to be 84.7%, 70.1%, 52.6%, 88.6%, and 81.6%, respectively. The SID of P in MCP, DCP, MSP, corn, and SBM were determined to be 91.8%, 76.8%, 94.4%, 73.1%, and 88.4%, respectively. The results of the present study indicate that the SID of Ca and P vary across different feed ingredients, indicating the types of ingredients that should be considered for accurate diet formulation.

## Figures and Tables

**Table 1:** Ingredient and chemical compositions of experimental diets on an as-fed basis.

Ingredient, g/kg	Ca–P-Free	MCP	DCP	MSP + Limestone	Non-Phytate P, 4.21g/kg		Ca/Total P, 1.4:1	
					SBM1	Corn1	SBM2	Corn2
Corn	–	–	–	–	–	810.00	–	810.00
Soybean meal	–	–	–	–	386.42	–	386.42	–
Sucrose	300.00	300.00	300.00	300.00	–	–	–	–
Cornstarch	455.37	435.91	433.13	418.32	539.63	58.54	548.77	67.44
Cellulose	50.00	50.00	50.00	50.00	–	–	–	–
Soybean oil	30.00	30.00	30.00	30.00	30.00	30.00	30.00	30.00
Dried egg albumen	90.00	90.00	90.00	90.00	–	–	–	–
Potassium carbonate	10.40	10.40	10.40	10.40	–	6.20	–	6.20
Sodium bicarbonate	1.00	1.00	1.00	–	–	–	–	1.00
Limestone	–	–	–	20.45	–	–	5.50	7.30
Monocalcium phosphate	–	19.80	–	–	–	–	–	–
Dicalcium phosphate	–	–	22.95	–	–	–	–	–
Monosodium phosphate	–	–	–	17.85	14.64	17.20	–	–
Magnesium oxide	0.90	0.56	0.19	0.65	–	–	–	–
Sodium chloride	4.00	4.00	4.00	4.00	4.00	4.00	4.00	4.00
Vitamin premix ^1^	1.50	1.50	1.50	1.50	1.50	1.50	1.50	1.50
Mineral premix ^2^	1.50	1.50	1.50	1.50	1.50	1.50	1.50	1.50
Choline chloride	2.00	2.00	2.00	2.00	2.00	2.00	2.00	2.00
Chromium oxide	5.00	5.00	5.00	5.00	5.00	5.00	5.00	5.00
L-Arg	8.64	8.64	8.64	8.64	1.19	9.81	1.19	9.81
L-His	3.51	3.51	3.51	3.51	0.41	3.85	0.41	3.85
L-Ile	4.17	4.17	4.17	4.17	1.05	6.11	1.05	6.11
L-Leu	6.46	6.46	6.46	6.46	1.00	6.58	1.00	6.58
L-Lys-HCl	8.44	8.44	8.44	8.44	2.51	12.27	2.51	12.27
L-Met	2.51	2.51	2.51	2.51	3.11	3.95	3.11	3.95
L-Cys	2.03	2.03	2.03	2.03	2.16	2.98	2.16	2.98
L-Phe	2.79	2.79	2.79	2.79	–	4.70	–	4.70
L-Thr	4.53	4.53	4.53	4.53	1.89	5.92	1.89	5.92
L-Trp	1.02	1.02	1.02	1.02	–	1.49	–	1.49
L-Val	4.23	4.23	4.23	4.23	1.99	6.40	1.99	6.40
Calculated value								
AME, kcal/kg	3708.6	3629.4	3618.1	3557.8	3226.0	3376.2	3263.2	3412.5
Crude protein	122.5	122.5	122.5	122.5	190.0	124.5	190.0	124.5
Calcium	0.26	3.87	5.11	7.50	1.35	0.24	3.30	2.83
Non-phytate phosphorus	0.10	4.21	4.21	4.21	4.21	4.21	0.84	0.25
Total P	0.10	4.21	4.21	4.21	5.73	5.98	2.36	2.03
Analyzed value								
Calcium	0.30	4.20	5.10	7.30	1.90	0.23	4.30	2.90
Total P	0.15	4.90	4.40	4.70	7.70	6.20	3.90	2.10

AME = apparent metabolizable energy; MCP = monocalcium phosphate; DCP = dicalcium phosphate; MSP = monosodium phosphate; SBM = soybean meal; P = phosphorus. ^1^ Supplies the following quantities per kilogram of diet: vitamin A, 2,400,000 IU; vitamin D_3_, 800,000 IU; vitamin E, 10,000 IU; vitamin K_3_, 640 mg; thiamin, 640 mg; riboflavin, 1720 mg; nicotinic acid, 12,000 mg; pantothenic acid, 4000 mg; pyridoxine, 900 mg; cobalamin, 4 mg; folacin, 440 mg; biotin 44 mg. ^2^ Supplies the following quantities per kilogram of diet: Mn, 24,000 mg; Zn, 22,000 mg; Fe, 10,000 mg; Co, 3200 mg; I, 250 mg; Se, 60 mg.

**Table 2:** Analyzed total calcium (Ca) and phosphorus (P) concentrations (%) of feed ingredients on an as-fed basis.

Ingredients	Total Ca	Total P
MCP	18.20	20.77
DCP	21.12	17.91
MSP	–	23.01
Limestone	35.41	–
Corn	0.03	0.25
SBM	0.35	0.61

MCP = monocalcium phosphate; DCP = dicalcium phosphate; MSP = monosodium phosphate; SBM = soybean meal.

**Table 3:** The apparent ileal digestibility (AID) and the standardized ileal digestibility (SID) of calcium (Ca) and phosphorus (P) in feed ingredients for broiler from chickens at day 21 ^1^.

Ingredients	AID of Ca	AID of P	SID ^2^ of Ca	SID of P
MCP	83.4 ^a^	90.5 ^a^	84.7 ^ab^	91.8 ^a^
DCP	69.0 ^b^	75.3 ^c^	70.1 ^c^	76.8 ^c^
MSP	–	93.0 ^a^	–	94.4 ^a^
Limestone	51.9 ^c^	–	52.6 ^d^	–
Corn	65.2 ^b^	70.2 ^d^	88.6 ^a^	73.1 ^d^
SBM	78.6 ^a^	86.8 ^b^	81.6 ^b^	88.4 ^b^
SEM ^3^	1.25	0.86	1.25	0.86
*p* values	<0.0001	<0.0001	<0.0001	<0.0001

MCP = monocalcium phosphate; DCP = dicalcium phosphate; MSP = monosodium phosphate; SBM = soybean meal. ^a–d^ Values with a different superscript within the column differ significantly (*p* < 0.05). ^1^ Data are presented as the least squares means of eight observations per treatment. ^2^ SID values were corrected for the ileal endogenous loss of Ca and P (58.98 and 66.37 mg/kg of dry matter intake, respectively). ^3^ SEM = standard error of the means.

## Data Availability

The original contributions presented in the study are included in the article, further inquiries can be directed to the corresponding author.
